# Supplementary material for: The human ribosome modulates multidomain protein biogenesis by delaying cotranslational domain docking
Source: Nat Struct Mol Biol. 2025 Sep 19;32(11):2296–307. doi: 10.1038/s41594-025-01676-5 (PMC12618258; doi:10.1038/s41594-025-01676-5)
Supplement: Supplementary file 1 — Supplementary Table 1: Cryo-EM data collection, map reconstruction and model refinement. [file 41594_2025_1676_MOESM1_ESM.pdf]

nature structural & molecular biology

Supplementary information https://doi.org/10.1038/s41594-025-01676-5

The human ribosome modulates multidomain protein biogenesis by delaying cotranslational domain docking

In the format provided by the authors and unedited

Supplementary Table 1. Cryo-EM data collection, map reconstruction and model refinement.

**EMDB**	EMD-51611
**PDB**	9GUL
**Data collection**	
Microscope	Titan Krios
Voltage (kV)	300
Magnification	130,000×
Electron exposure ($e^-/Å^2$)	40.8
Defocus range (µm)	-0.5 to -2.3
Detector	Falcon 4i with Selectris energy filter
Pixel size (Å)	0.95
Movies recorded	35,616
**Reconstruction**	
Particles	788,554
Map resolution (Å) (FSC threshold = 0.143)	2.2
Map resolution range (Å)	2.0 – 4.3
**Model refinement**	
Model resolution (Å) (FSC threshold = 0.5)	2.2
Number of residues	
Protein	6464
Nucleic acid	3949
Water	94
Ligand	288
B factors ($Å^2$)	
Protein	85.00
Nucleic acid	106.69
Water	66.45
Ligand	67.15
RMS deviations	
Bond lengths (Å)	0.004
Bond angles (°)	0.635
**Validation**	
MolProbity score	1.29
Clash score	2.99
Rotamer outliers (%)	1.12
Ramachandran plot	
Favoured (%)	97.09
Allowed (%)	2.91
Outliers (%)	0
