# Supplementary material for: The human ribosome modulates multidomain protein biogenesis by delaying cotranslational domain docking
Source: Nat Struct Mol Biol. 2025 Sep 19;32(11):2296–307. doi: 10.1038/s41594-025-01676-5 (PMC12618258; doi:10.1038/s41594-025-01676-5)
Supplement: Supplementary file 2 — Reporting Summary [file 41594_2025_1676_MOESM2_ESM.pdf]

nature portfolio

Corresponding author(s): David Balchin

Last updated by author(s): Sep 19, 2024

Reporting Summary

Nature Portfolio wishes to improve the reproducibility of the work that we publish. This form provides structure for consistency and transparency in reporting. For further information on Nature Portfolio policies, see our Editorial Policies and the Editorial Policy Checklist.

Statistics

For all statistical analyses, confirm that the following items are present in the figure legend, table legend, main text, or Methods section.

n/a	Confirmed	
☐	☒	The exact sample size (*n*) for each experimental group/condition, given as a discrete number and unit of measurement
☐	☒	A statement on whether measurements were taken from distinct samples or whether the same sample was measured repeatedly
☐	☒	The statistical test(s) used AND whether they are one- or two-sided<br>*Only common tests should be described solely by name; describe more complex techniques in the Methods section.*
☒	☐	A description of all covariates tested
☒	☐	A description of any assumptions or corrections, such as tests of normality and adjustment for multiple comparisons
☐	☒	A full description of the statistical parameters including central tendency (e.g. means) or other basic estimates (e.g. regression coefficient) AND variation (e.g. standard deviation) or associated estimates of uncertainty (e.g. confidence intervals)
☐	☒	For null hypothesis testing, the test statistic (e.g. *F*, *t*, *r*) with confidence intervals, effect sizes, degrees of freedom and *P* value noted<br>*Give P values as exact values whenever suitable.*
☒	☐	For Bayesian analysis, information on the choice of priors and Markov chain Monte Carlo settings
☒	☐	For hierarchical and complex designs, identification of the appropriate level for tests and full reporting of outcomes
☒	☐	Estimates of effect sizes (e.g. Cohen's *d*, Pearson's *r*), indicating how they were calculated

Our web collection on statistics for biologists contains articles on many of the points above.

Software and code

Policy information about availability of computer code

Data collection	HDX-MS data were collected using a Synapt G2Si (Waters). Proteomics data were collected using a Lumos Tribrid Orbitrap.<br>CryoEM data were collected using EPU v3.8.1.7603 (ThermoFisher Scientific) on a Titan Krios equipped with a Falcon 4i detector and Selectris energy filter.
Data analysis	PLGS 3.0 (Waters), Dynamx 3.0 (Waters) and HXexpress were used for analysis of HDX-MS data. Proteomics data were analysed using Maxquant 2.5 and Perseus 1.6.2.3. Western blots were analysed in ImageJ. Other data were analysed using Graphpad Prism 9. CryoEM initial particle picking, reconstruction, classification and refinement in Cryosparc v4.4.1. Further refinement in Relion 5. Model building using WinCoot v0.9.8.93, Isolde v1.7.1 and ChimeraX v1.7.1. Model refinement in Phenix v1.21.

For manuscripts utilizing custom algorithms or software that are central to the research but not yet described in published literature, software must be made available to editors and reviewers. We strongly encourage code deposition in a community repository (e.g. GitHub). See the Nature Portfolio guidelines for submitting code & software for further information.

Data

Policy information about availability of data

All manuscripts must include a data availability statement. This statement should provide the following information, where applicable:

- Accession codes, unique identifiers, or web links for publicly available datasets
- A description of any restrictions on data availability
- For clinical datasets or third party data, please ensure that the statement adheres to our policy

All mass spectrometry data have been deposited to the ProteomeXchange Consortium via the PRIDE partner repository with the dataset identifiers PXD055069; PXD055060; PXD055251; PXD055280; PXD055476. Proteomic analysis used the Uniprot E. coli reference proteome (UP000000625).
The electron density reconstructions and structure coordinates were deposited to the Electron Microscopy Database (EMDB) and to the PDB under the following accession codes: EMD-51611 and PDB: 9GUL. Structural models used to initiate model building were accessed from the PDB: PDB 6QZP and PDB 6R5Q.

Research involving human participants, their data, or biological material

Policy information about studies with human participants or human data. See also policy information about sex, gender (identity/presentation), and sexual orientation and race, ethnicity and racism.

Reporting on sex and gender	Not applicable
Reporting on race, ethnicity, or other socially relevant groupings	Not applicable
Population characteristics	Not applicable
Recruitment	Not applicable
Ethics oversight	Not applicable

Note that full information on the approval of the study protocol must also be provided in the manuscript.

Field-specific reporting

Please select the one below that is the best fit for your research. If you are not sure, read the appropriate sections before making your selection.

☒ Life sciences ☐ Behavioural & social sciences ☐ Ecological, evolutionary & environmental sciences

For a reference copy of the document with all sections, see nature.com/documents/nr-reporting-summary-flat.pdf

Life sciences study design

All studies must disclose on these points even when the disclosure is negative.

Sample size	No statistical methods were used to calculate sample sizes. Biochemical experiments were repeated at least three times, and in some cases using independent protein purifications. 3-5 replicates of HDX-MS data were collected to account for technical variability in pipetting, considering prior evidence in the literature supporting the high technical reproducibility of these experiments, and consistent with community guidelines for HDX MS (Masson, G. R. et al. Recommendations for performing, interpreting and reporting hydrogen deuterium exchange mass spectrometry (HDX-MS) experiments. Nat Methods 16, 595–602 (2019)). In some cases, replicate data were also acquired for independent purifications of the protein complexes to assess biological variability. For other experiments (PK assays, Cys labelling), the high reproducibility and large effect sizes indicate that the number of replicates was sufficient. For structural studies, 788,554 particles were picked from 29,080 movies. No sample size calculation was performed for the cryoEM data; the sample size is sufficient for the resolution of the map reported here.
Data exclusions	For structural studies, electron micrograph movies with substantial drift and crystalline ice were excluded. This is a pre-established standard in the cryoEM community. Picked particles were excluded based on 2D and 3D classification. Excluded particles were those belonging to classes that lacked high-resolution structural features; this is a pre-established standard in the cryoEM community.<br>No other data were excluded.
Replication	All experiments except for structure determination were confirmed using replicate measurements. CryoEM single particle analysis involves averaging many independent particles.
Randomization	No randomization was performed, as samples were not grouped.
Blinding	No blinding was performed, as samples were not grouped. Moreover, MS data were analysed using standard pipelines which minimise the possibility of subjective interpretation.

Reporting for specific materials, systems and methods

We require information from authors about some types of materials, experimental systems and methods used in many studies. Here, indicate whether each material, system or method listed is relevant to your study. If you are not sure if a list item applies to your research, read the appropriate section before selecting a response.

Materials & experimental systems

n/a	Involved in the study
☐	☒ Antibodies
☐	☒ Eukaryotic cell lines
☒	☐ Palaeontology and archaeology
☒	☐ Animals and other organisms
☒	☐ Clinical data
☒	☐ Dual use research of concern
☒	☐ Plants

Methods

n/a	Involved in the study
☒	☐ ChIP-seq
☒	☐ Flow cytometry
☒	☐ MRI-based neuroimaging

Antibodies

Antibodies used	Anti-FLuc (Abcam, ab185923, lot# GR3317915-2). Diluted 1:1000
Validation	DOI: 10.1093/nar/gkab1241<br><br>Also validated in the current manuscript using purified FLuc.

Eukaryotic cell lines

Policy information about cell lines and Sex and Gender in Research

Cell line source(s)	Expi293F suspension cells (ThermoFisher)
Authentication	The cell line was not authenticated
Mycoplasma contamination	The cell line tested negative for mycoplasma
Commonly misidentified lines<br>(See ICLAC register)	*Name any commonly misidentified cell lines used in the study and provide a rationale for their use.*

Plants

Seed stocks	*Report on the source of all seed stocks or other plant material used. If applicable, state the seed stock centre and catalogue number. If plant specimens were collected from the field, describe the collection location, date and sampling procedures.*
Novel plant genotypes	*Describe the methods by which all novel plant genotypes were produced. This includes those generated by transgenic approaches, gene editing, chemical/radiation-based mutagenesis and hybridization. For transgenic lines, describe the transformation method, the number of independent lines analyzed and the generation upon which experiments were performed. For gene-edited lines, describe the editor used, the endogenous sequence targeted for editing, the targeting guide RNA sequence (if applicable) and how the editor was applied.*
Authentication	*Describe any authentication procedures for each seed stock used or novel genotype generated. Describe any experiments used to assess the effect of a mutation and, where applicable, how potential secondary effects (e.g. second site T-DNA insertions, mosiacism, off-target gene editing) were examined.*
