# Supplementary material for: The human ribosome modulates multidomain protein biogenesis by delaying cotranslational domain docking
Source: Nat Struct Mol Biol. 2025 Sep 19;32(11):2296–307. doi: 10.1038/s41594-025-01676-5 (PMC12618258; doi:10.1038/s41594-025-01676-5)

Pellowe et al. The human ribosome modulates multidomain protein biogenesis by delaying cotranslational domain docking

Figure 1D and E – hRNC gels

Green box shows cropped region in uL2 blot. The minor band in this blot is signal for eS24 – a protein within the removed 40S subunit.

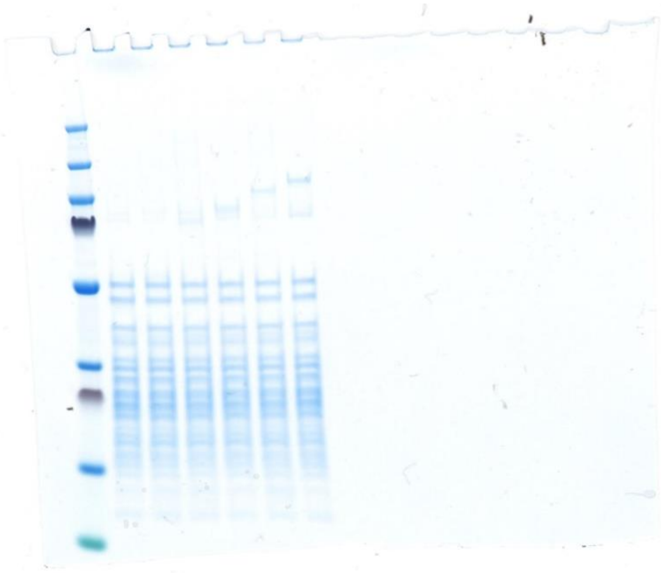

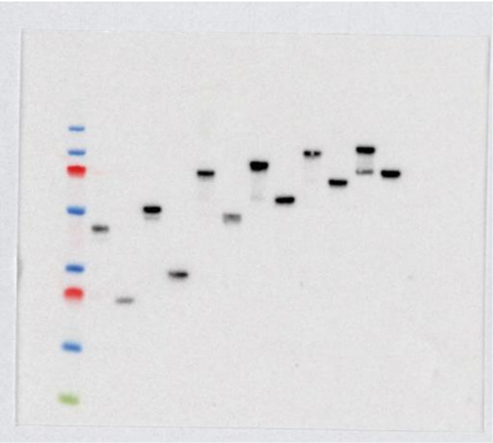

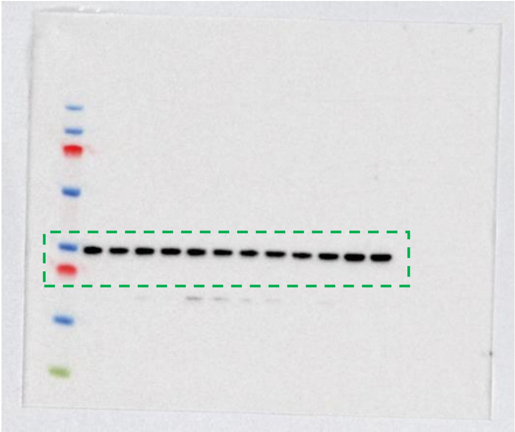

Figure S1A – Xbp1u vs Xbp1u+ stalling efficiency

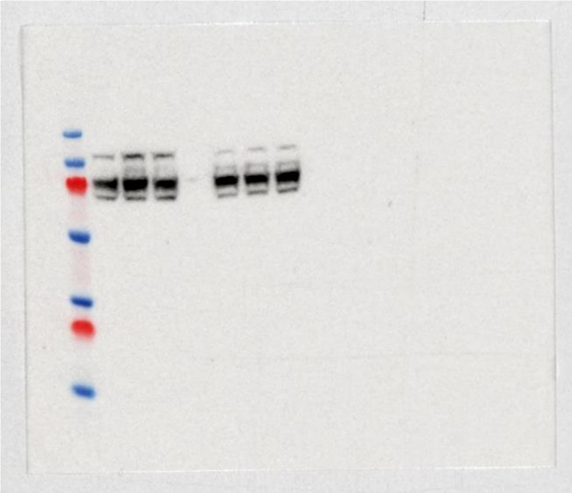

Figure S1B – Post-activity Western blot for hRNCs

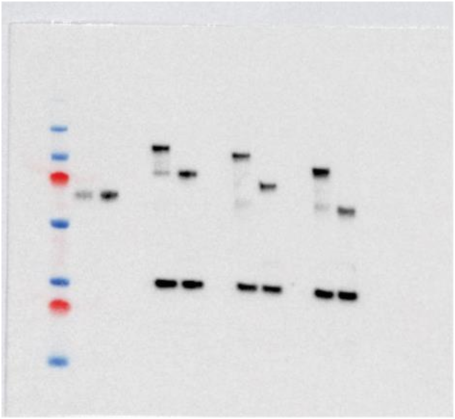

Figure 4A – ProteinaseK digests of RNCs

Green boxes show data included in figure. The right-hand side of each blot is a different experiment which was not included in this manuscript.

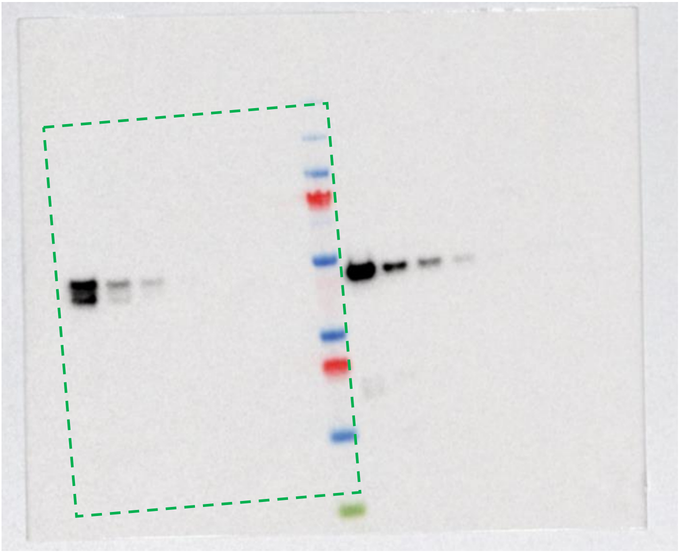

Ns-RNC

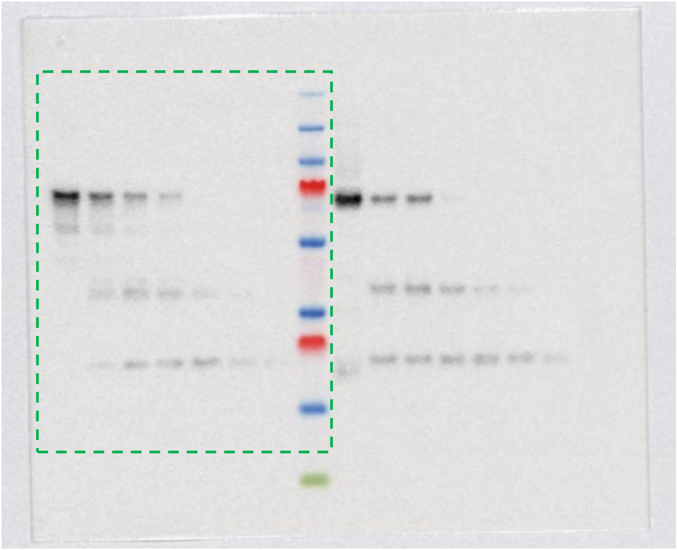

1/2N-RNC

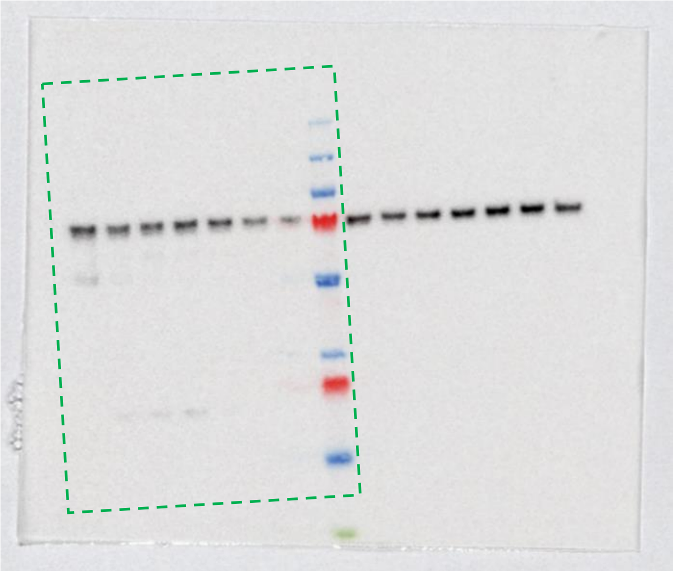

N-RNC

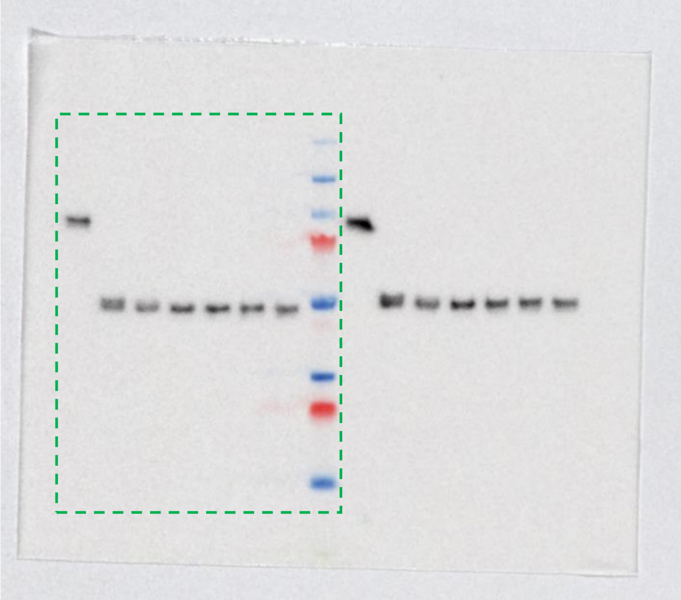

T-RNC

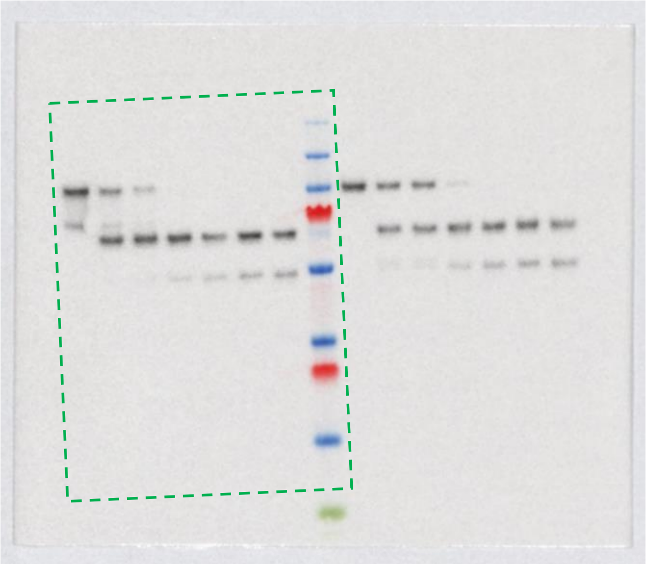

FL-RNC

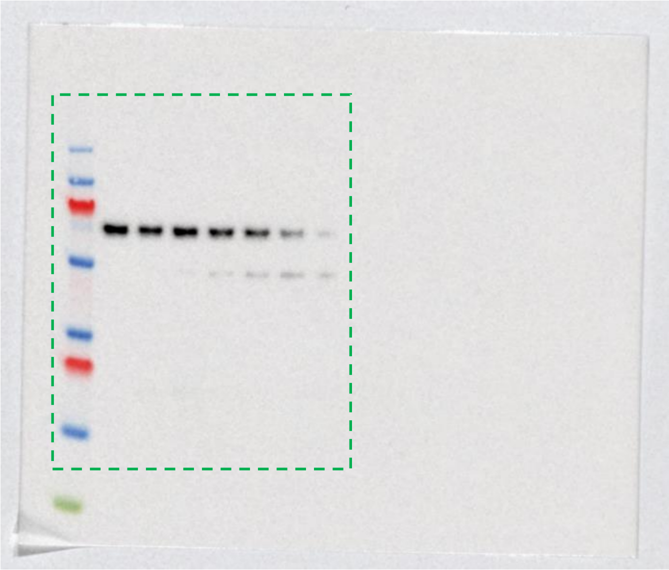

FL-FLuc

Figure S6B – Isolated Ns Coomassie

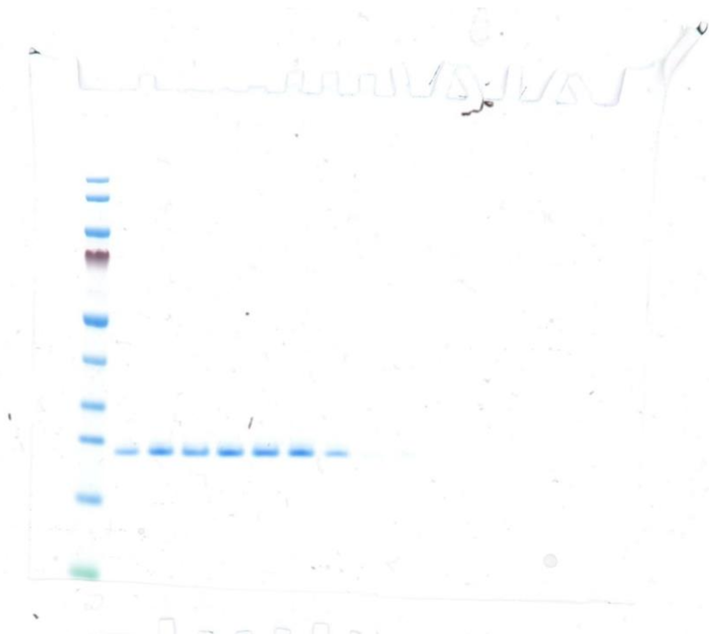

Figure S7B – F5M cysteine labelling

Uncropped example gel. Green boxes show cropped area.

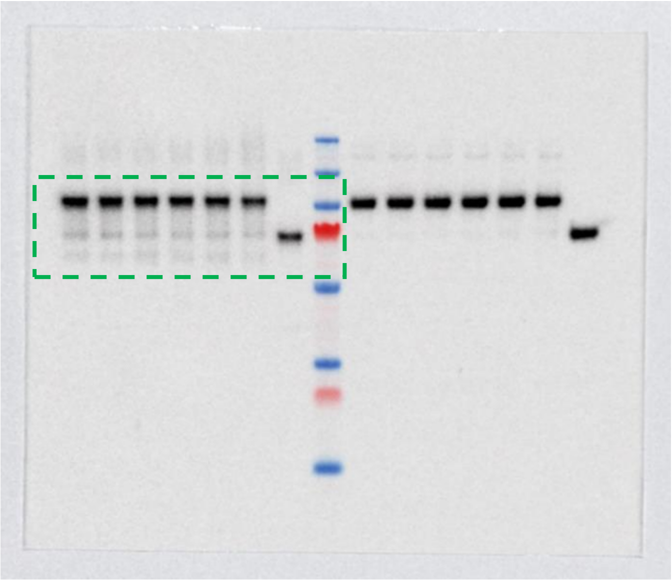

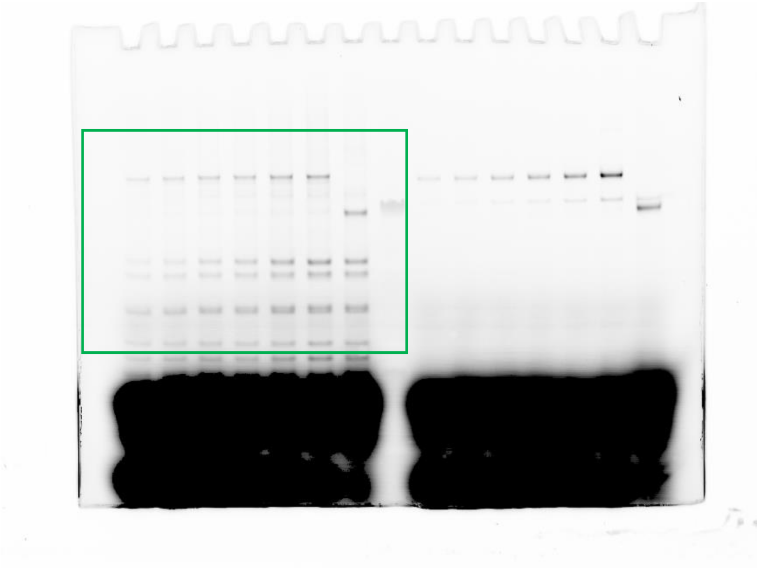

Figure S7C & D – F5M cysteine labelling raw data

Fluorescence gels from Typhoon 9500. Green arrow shows quantified band.

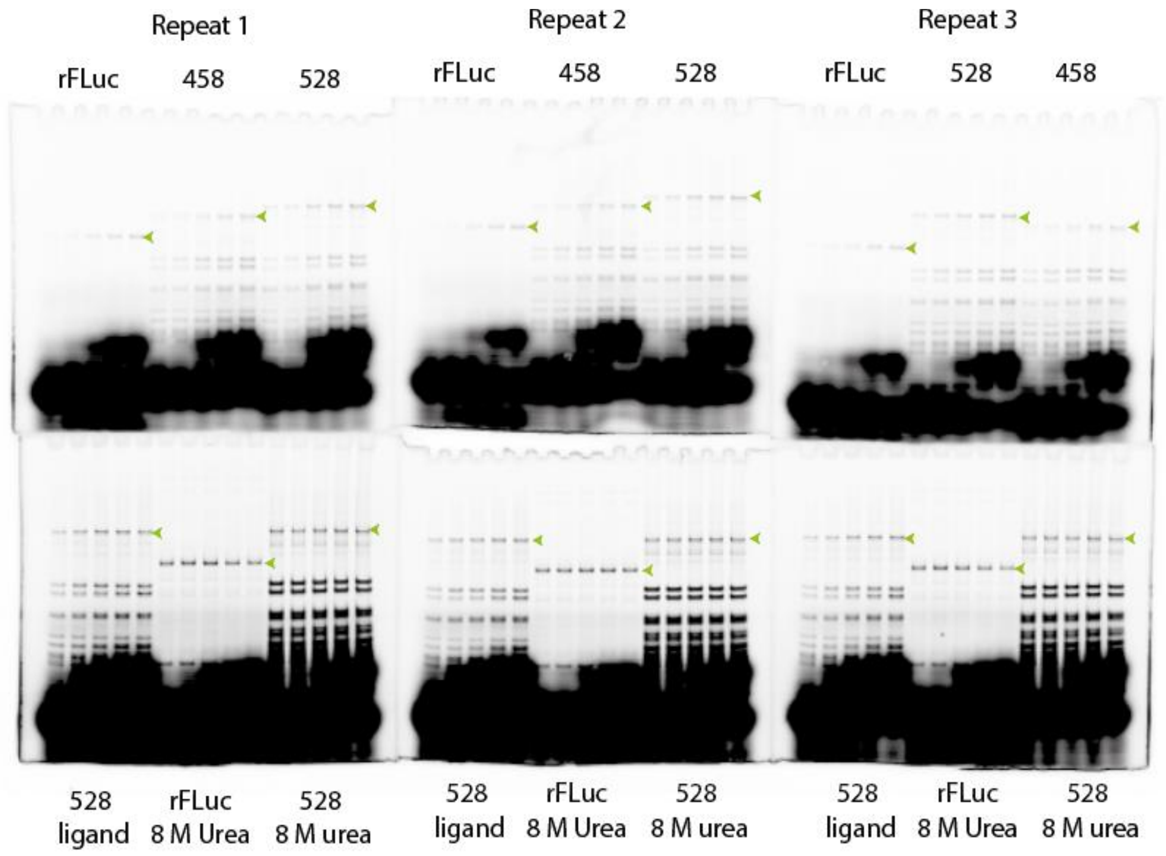

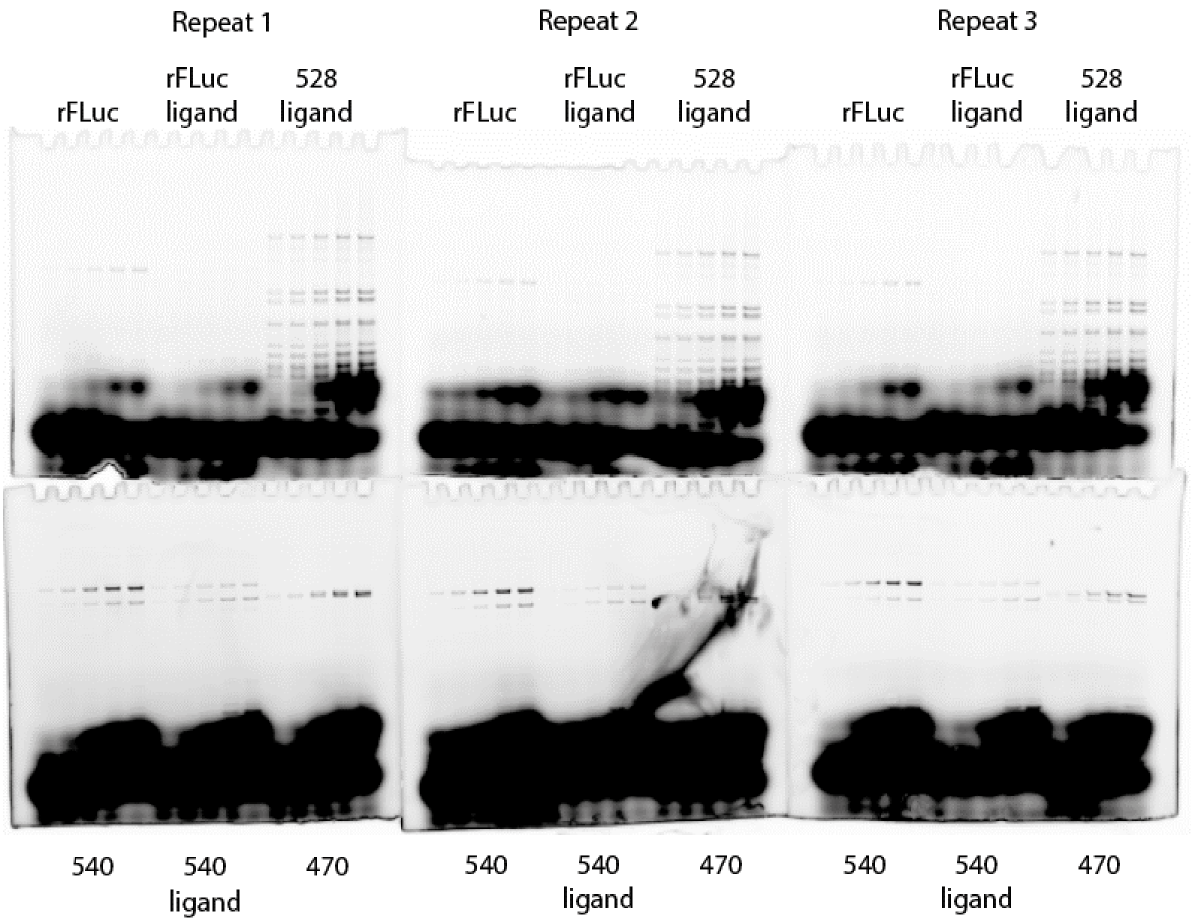
Repeat 1
Repeat 2
Repeat 3
rFLuc
rFLuc ligand
528 ligand
rFLuc
rFLuc ligand
528 ligand
rFLuc
rFLuc ligand
528 ligand
540
540 ligand
470
540
540 ligand
470
540
540 ligand
470

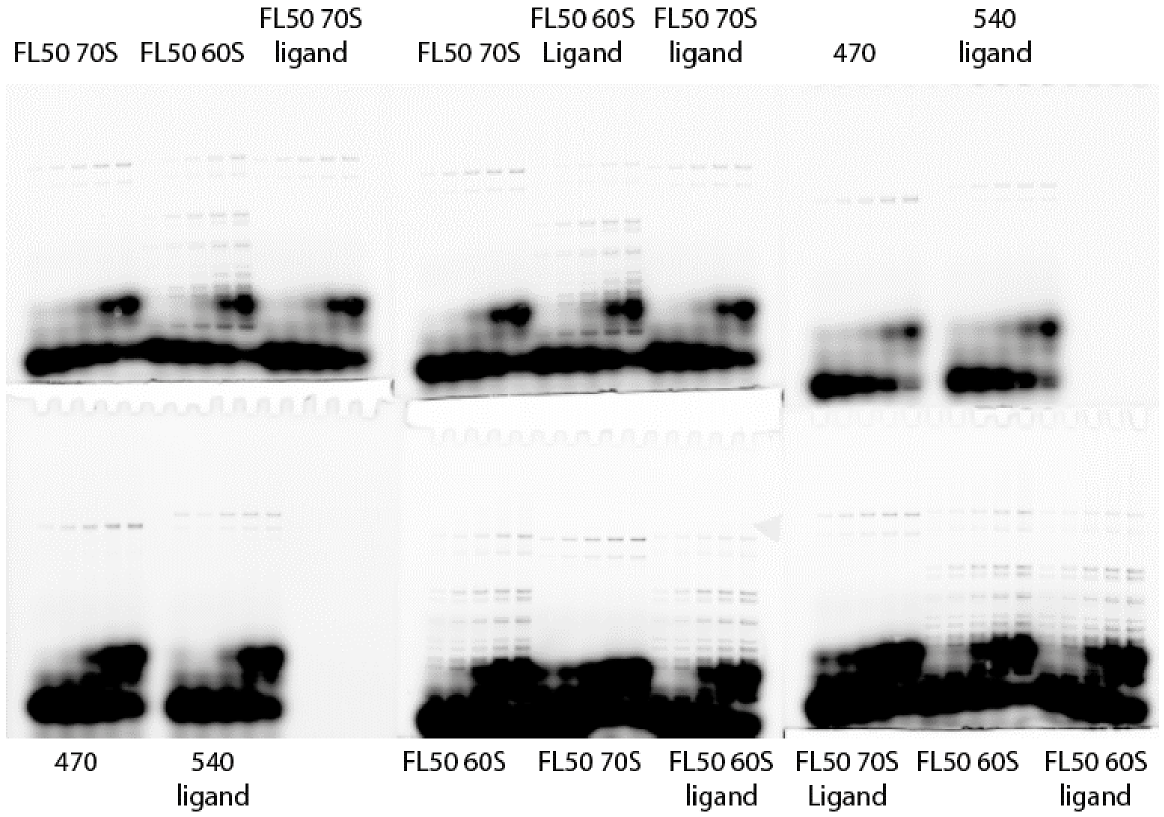
FL50 70S
FL50 60S
FL50 70S ligand
FL50 70S
FL50 60S Ligand
FL50 70S ligand
470
540 ligand
470
540 ligand
FL50 60S
FL50 70S
FL50 60S ligand
FL50 70S Ligand
FL50 60S
FL50 60S ligand

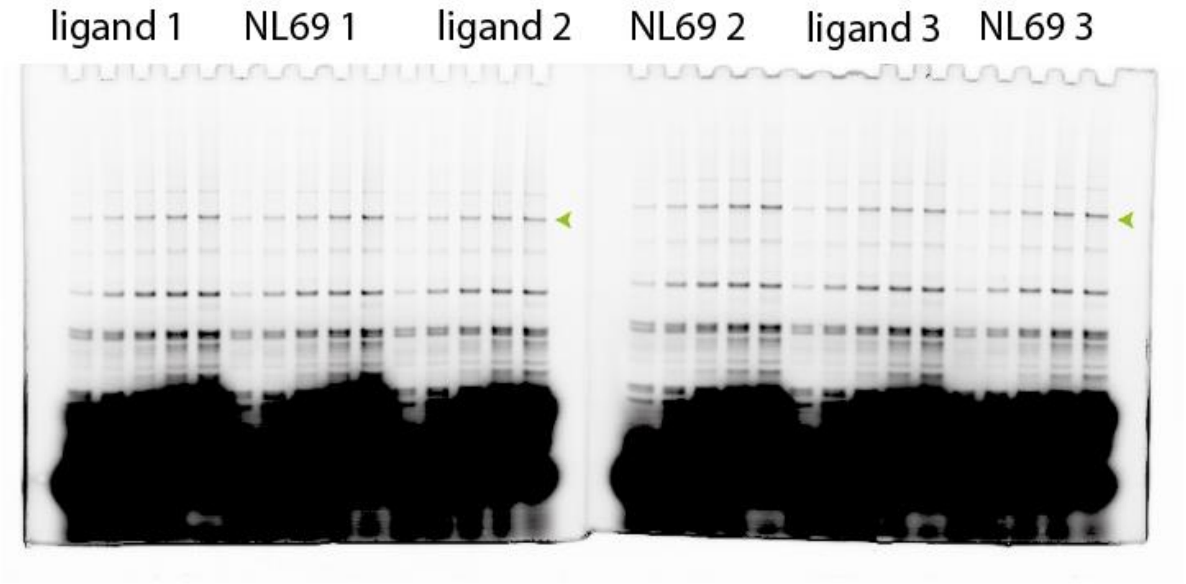

Figure S8A & B – bRNC Western blots and Coomassie

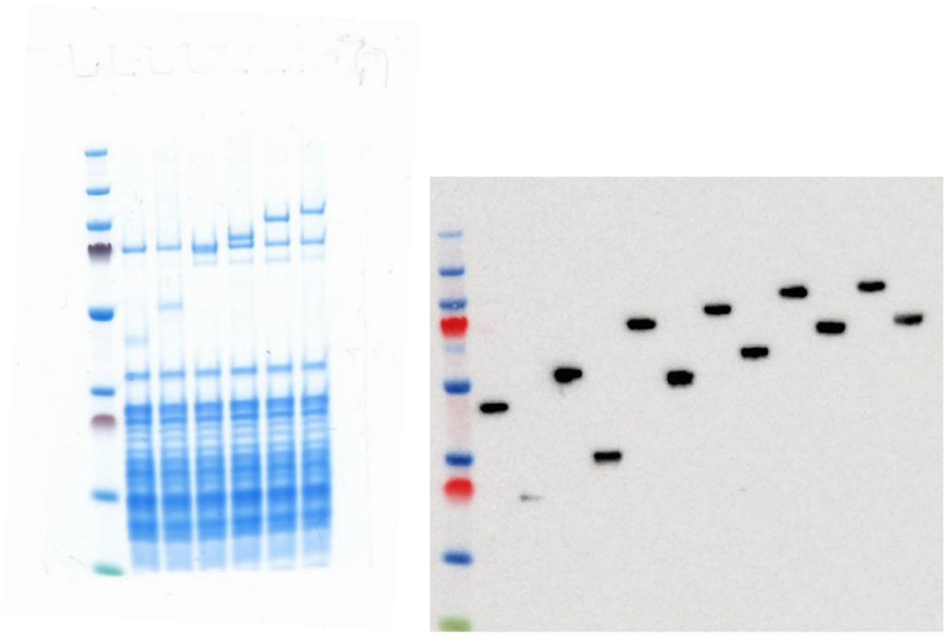

Supplement: Supplementary file 12 — Combined unmodified gels and blots. [file 41594_2025_1676_MOESM12_ESM.pdf]
